# Novel dermis niche equivalent based on a 3D-structure fabricated by 2-photon polymerization

**DOI:** 10.1016/j.bbiosy.2026.100143

**Published:** 2026-07-23

**Authors:** Sebastian Schröder, Holger Rothe, Elisabeth Rehbein-Bode, Doris Heinrich, Nicole Teusch, Nadine Brandes

**Affiliations:** a Institute for Bioprocessing and Analytical Measurement Techniques e.V. (iba), Heilbad Heiligenstadt, Germany; b Institute of Pharmaceutical Biology and Biotechnology, Faculty of Mathematics and Natural Science, Heinrich Heine University Düsseldorf, Duesseldorf, Germany; c Institute of Chemistry and Bioengineering, Faculty of Mathematics and Natural Science, Technical University Ilmenau, Ilmenau, Germany

**Keywords:** Skin topography, 2-Photon polymerization, Tissue engineering, 3D-printing, GelMA, CAD, Human neonatal dermal fibroblasts

## Abstract

The skin as the largest human organ serves several functions, such as natural protection against internal as well as external environmental influences. Three-dimensional (3D)-tissue engineering has emerged as a promising approach for the development of functional and physiologically relevant dermis substitutes. Mimicking the topography of the dermis to develop a skin-niche model (SKN) that contains epidermal stem cells is still a challenge. This study aimed to develop a dermis equivalent featuring a physiologically relevant dermal-epidermal junction (DEJ) topography; hence, a pattern was printed using 2-photon polymerization (2PP). The depth of the invaginations can influence cell differentiation and proliferation of epidermal stem cells. The template was subsequently translated into a dermis equivalent (DE). The human neonatal dermal fibroblasts (HDFn) remodeled the DE over 14 days, resulting in reduced dimensions that remained within the physiological range. The DE contains gelatin methacrylate (GelMA) and proliferating fibroblasts that produce extracellular matrix (ECM). In a proof-of-concept, N/TERT-1 keratinocytes, as the epidermal layer, were seeded on the patterned DE to verify the adhesion and proliferation of keratinocytes on the DE. Overall, this approach enables the generation of a DE with a natural DEJ, as a more physiological skin model for future use in disease modeling, drug development, screening, and skin transplantation.

## Highlights

• Two-photon polymerization enabled precise fabrication of DEJ-like templates.
• A molding strategy transferred micron-scale features into GelMA hydrogels.
• Fibroblasts generated an ECM-rich dermis equivalent with physiological topography.
• Tissue remodeling identified an optimal rete ridge architecture (P7).
• The model supports epidermal attachment and basement membrane formation.

## Graphical abstract

**Figure:**
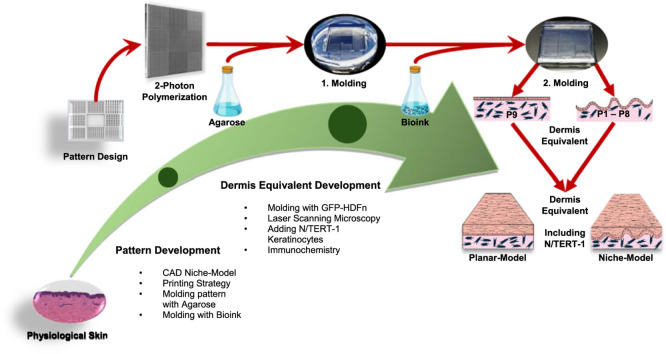

## 1 Introduction

**Fig. 1:**
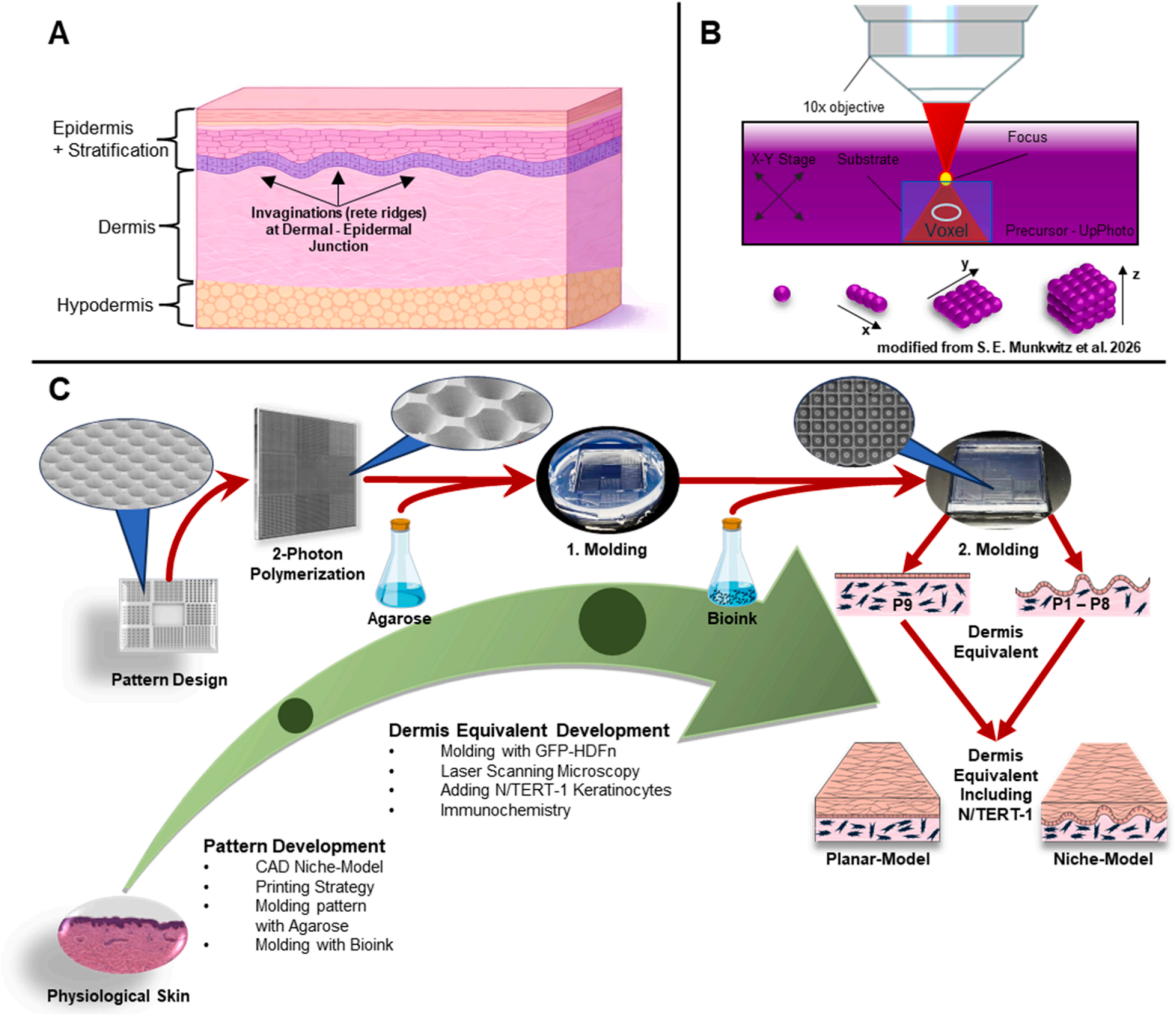
A Simplified diagram of the skin including epidermis, dermis, and part of the hypodermis. The focus is on the dermal-epidermal junction with its natural topography. Basal epidermal keratinocytes adhere directly to the structured dermis and form the direct connection between the dermis and the epidermis. The epidermis stratifies from this level to the outermost stratum corneum. B Principles of 2-photon polymerization [16]. A pulsed infrared laser is focused through a microscope objective, causing two photons to hit the photo initiator simultaneously at the focal point, the so-called voxel, thereby providing the energy required to activate the photo initiator causing polymerization. Polymerization is not initiated in areas not in focus, resulting in very high resolution. C From pattern development (CAD pattern design, printing methods and translation to a hydrogel) to the dermis equivalent development (seeding fibroblasts in GelMA pattern, analyzing cell proliferation and spreading, and finally seeding N/TERT-1 keratinocytes on the dermis equivalent analyzed by immunohistochemistry) including keratinocytes.

The skin is divided into three main layers, the epidermis, dermis and hypodermis; and protects the organism from internal and external influences as ultraviolet (UV) light, pathogens and mechanical injuries [1]. The dermis and epidermis are highly involved in the wound healing process when the skin surface is damaged by cuts and burns. Fibroblasts are mainly located in the dermis whereas the main cell type of the epidermis is the keratinocyte [1]. These two layers are structurally and functionally connected by the dermal epidermal junction (DEJ), a highly specialized interface that mediates mechanical stability, biochemical signaling and epidermal homeostasis [2]. Self-renewal of the epidermis is achieved through activation of epidermal stem cells [3]. These epidermal stem cells are localized mainly in the base or on the plateaus of the invaginations, so-called “rete ridges”, depending on the location of the skin and on age [4]. The rete ridges, in healthy skin are between 80 – 200 µm in depth, 50 – 450 µm in width and vary based on location and skin type (Fig. 1 A) [5,6]. On the palm of the hands and on the soles of the feet, epidermal stem cells are bundled in the base of the rete ridges*,* whereas on the rest of the body they are localized on the plateaus of the rete ridges [4]. During the ageing process, the number of epidermal stem cells and the depth of the rete ridges decreases, which leads to slower healing, a smaller surface area and thus reduced tear resistance [7]. In addition, the production of extracellular matrix (ECM) molecules such as collagen VII and collagen IV, as well as adhesion proteins in the DEJ such as laminin-332, collagen XVII, and integrin α6β4, decreases with age [8]. Laminin-332 and collagen IV (ColIV) are essential components of the skin’s basement membrane, where they not only ensure structural integrity but also mediate stable adhesion between the dermis and epidermis through their crucial role in the formation of hemidesmosomes with collagen XVII [9]. In this way, they regulate the proliferation, migration, and differentiation of keratinocytes and ultimately contribute to the maintenance of skin homeostasis and barrier function. Skin diseases such as dystrophic epidermolysis bullosa, which causes reduced depth of the rete ridges, results in poor adhesion of the epidermis to the dermis, which in turn leads to blister formation [10]. Other diseases such as psoriasis vulgaris are characterized by markedly elongated rete ridges [11]. During the proliferation phase in wound healing, dermal fibroblasts differentiate into myofibroblasts, which increases the proliferation and ECM synthesis rate [12]. Additionally, myofibroblasts contract the wound edges and pull them together, using a mechanism similar to smooth muscle cells [13]. Myofibroblasts deposit fibronectin, one of the major extracellular matrix proteins, thereby providing a provisional extracellular matrix that supports keratinocyte adhesion and contributes to subsequent basement membrane formation [14,15].

Conventional flat skin models, while providing a basic framework for studying skin biology, often overlook critical features such as rete ridges, hair follicles, and sebaceous glands [[17], [18], [19]]. These structures are important for several reasons: they contribute to the skin’s barrier function, facilitate cell-to-cell interactions, and play a vital role in the skin's immune response and overall homeostasis [[20], [21], [22]]. Today, there are various skin models commercialized, which include the dermis and epidermis but do not consider in full the above-mentioned properties [23]. For example Phenion® [19], Episkin [18] and EpiDerm™ [17] are used in basic research e.g. adding immune cells to the Phenion® model [24,25], for drug development [26] and for skin cosmetics [17,27]. Still, none of these skin models has a physiological topography of the dermal epidermal junction (DEJ) for better predictions and increased regeneration rates. However, there are some innovative approaches to generate skin topographies in literature using three-dimensional (3D)-bioprinting, spinning or stamp methods [[28], [29], [30]]. Yet, conventional DLP and extrusion printers are severely limited in their resolution and cannot fully replicate the physiological topography [31]. But in recent years, 2-photon polymerization (2PP) has evolved from a niche technology to an emerging tool for the development and manufacturing of matrices for numerous applications in tissue engineering [32,33]. 2PP is a nonlinear laser lithography technique in which a tightly focused, femtosecond pulsed, near-infrared laser induces polymerization only within a sub-diffraction-limited focal volume (voxel) via simultaneous absorption of two photons by a photo initiator (Fig. 1 B) [16]. 2PP enables the fabrication of highly accurate 3D structures with sub-micrometer architectural features and therefore represents a promising approach for generating physiological dermal-epidermal junction topographies. This technique enables the production of complex surfaces such as the formation of rete ridges, which are essential for physiological processes in the skin [34]. Previous studies demonstrated the possibility to vary 2PP-written structure dimensions with high precision for different patterns, which can be adapted for developing a physiological DE [35].

Previous studies have highlighted the importance of epidermal stem cell-associated markers, including β1-integrin [[36], [37], [38]], as well as basement membrane proteins such as laminin-332 and ColIV for maintaining skin homeostasis [2,8,9]. Incorporating these biological features together with a physiologically relevant DEJ topography represents an important objective in the development of advanced skin models. The ability to tailor rete ridge geometry may facilitate the development of disease- and age-specific skin models. In drug research, a more physiologically relevant model can better predict the skin's response to therapeutic agents, allowing the effect of the substance to be studied in more detail in comparison to flat skin models [27]. The aim of this study was to develop a 2PP-printed physiological pattern template, which can be translated into a DE including human dermal fibroblasts and a physiological topography of the DEJ using gelatin methacrylate (GelMA) as ECM. Moreover, as a proof-of-concept, the DE is seeded with an immortalized epidermal keratinocyte layer to verify the potential of the recently developed 3D-topography (Fig. 1 C).

## 2 Materials and methods

### 2.1 Hydrogel preparation

Sterile OkaGel^TM^ (GelMA) solid (gel strength = 300 g Bloom, GelMA Technologies) is a gelatin-methacrylate with an 80% degree of substitution which was used for the fabrication of the patterned dermis equivalent (DE). GelMA solid was diluted to a stock solution of 15% (w/v) in sterile 1x Dulbecco’s phosphate-buffered saline (DPBS, Pan Biotech) and heated to 50°C until the complete GelMA was dissolved. From this stock solution, the working solutions of 10% and 5% GelMA were prepared including 2 mg/ml lithium phenyl (2,4,6-trimethylbenzoyl) phosphinate (LAP, Merck) from a stock solution of 40 mg/ml LAP diluted in DPBS. To prepare the LAP stock solution, the powder was diluted in preheated DPBS at 40°C and then sterilized by filtration.

Low melting agarose (Merck) was prepared in a 3% (w/v) solution in DPBS and sterilized by autoclaving. The agarose was used for sealing the Alvetex® scaffolds (described in 2.5) and molding the 2-photon polymerization (2PP) printed patterns to generate agarose beds (described in 2.6).

### 2.2 Pattern design and printing

The patterns were designed in Computer Aided Design (CAD, Autodesk Inventor Professional 2024), sliced in Think3D (UpNano), and printed with the NanoOne 2PP printer (UpNano) and UpPhoto (UpNano) as printing material, a ready-to-use precursor for 2PP. The standard settings for the 10x objective were used which cause a printing time of nearly 5 hours to print one screening pattern. As substrate the printing standard platform from UpNano was used. New printed constructs were washed first in propylene glycol 1-monomethyl ether 2-acetate (PGMEA, TCI Chemicals) and afterwards with isopropyl alcohol (Robbyrob). After washing the constructs, they were placed in a sterile petri dish and dried at 37°C overnight.

### 2.3 Gold sputtering and electron scanning microscopy

Before the printed patterns could be analyzed in the scanning electron microscopy (SEM, Evo LS 10, Zeiss) they were coated with a 150 nm gold layer using an EM ACE600 Goldsputter (LEICA). Each single gold coated pattern was analyzed in high vacuum mode using SE-detector for imaging. In the SEM analysis 45° angles were used for the overview pictures of each single pattern.

### 2.4 Cell culture

Green fluorescent protein-human neonatal dermal fibroblasts (GFP-HDFn, Angio-proteomie) and the cell line N/TERT-1 keratinocytes (Radboud University Medical Center) of immortalized human epidermal keratinocytes were used [39]. GFP-HDFn were cultured in Dulbecco’s modified eagle medium (DMEM) high glucose (Thermo Fisher Scientific Inc.) plus 10% (v/v) fetal calf serum (FCS, Pan Biotech) and 1% (v/v) penicillin-streptomycin (p/s, Gibco^TM^). N/TERT-1 keratinocytes were cultured in CnT-prime epithelial proliferation medium (CnT-PR, CELLnTEC) including 1% p/s. Both cell lines were cultured under standard conditions (5% CO_2_, 37°C). The cell culture medium was changed each second day. The GFP-HDFn were split when they were confluent and the N/TERT-1 keratinocytes when 90 - 100% confluency was reached. Both cell lines were split in a 1:3 ratio. TrypLE™ Express Enzyme (ThermoFisher Scientific) was used to detach cells from cell culture flasks.

### 2.5 GFP-HDFn proliferation assay in GelMA

The analysis of the spreading, proliferation and extracellular matrix production of GFP-HDFn in 5% (w/v) and 10% (w/v) GelMA was examined as follows. First Alvetex® scaffolds (AMSBIO) were treated with 80% ethanol, then washed three times with DPBS to activate the scaffold. Then 150 µl 3% (w/v) agarose was used to seal the scaffolds. To ensure that, no GelMA can interfere with the scaffold. The GFP-HDFn were added to the prepared GelMA and mixed by careful pipetting. 150 µl of each sample was added to the sealed Alvetex® scaffolds, instantly photocured and then incubated in prepared DMEM at 37°C for 1 - 14 days. Subsequently, the samples were fixed with 4% (w/v) paraformaldehyde and detached from the Alvetex® scaffolds. This procedure helps to avoid GFP-HDFn adhesion to other materials and the GFP-HDFn interaction with GelMA can be examined. Additionally, at day 7 and 14 the samples were stained and analyzed with confocal laser scanning microscopy 710 (cLSM, Zeiss) as described in 2.7. For the quantitative analysis of the fluorescence data, the mean fluorescence of the images (GFP-HDFn and fibronectin-AF647) was determined. To do this, the contrast of the images was adjusted to identical values, and the images were then analyzed in three different ways using ImageJ, from which the average was calculated. In the first run, the thresholds were determined automatically using ImageJ; in the second, a fixed point was selected where the weakest signal of all images was clearly visible; and in the third, the threshold was set based on own assessment.

### 2.6 Skin substitute preparation

For that purpose, the 2PP-printed pattern templates were molded with 3% (w/v) low melting agarose then the 2PP-printed pattern templates were detached. A mixture of GelMA and GFP-HDFn with an end concentration of 750,000 cells/ml and at passage number ≤10 and 5% (w/v) GelMA was prepared for generating scaffold patterns. The prepared bioink (5% GelMA including GFP-HDFn) was added onto the remaining agarose bed, photocured (Omnicure3000) at 405 nm wavelength, with 50% power and with a distance to the sample of 40 mm for 10 s. Some DPBS was added to the sample (to keep the hydrogel wet) and the GelMA pattern was detached from the agarose bed by cutting the edges of the agarose bed and flipping the whole sample. Afterwards the remains of the agarose bed can be removed and the GelMA pattern remains undamaged. With an in-house-designed spoon the dermis equivalents (DE) can be simply transferred to clean 6-well plates. Then the DEs were cultured for 10 days in DMEM plus 10% (v/v) FCS and 1% (v/v) p/s. For the last four days, the DE was cultivated in CnT-Prime epithelial/stromal co-culture medium (CnT PR-CC, CELLnTEC). Subsequently 500,000 N/TERT-1 keratinocytes were added onto the pattern. The skin models were cultured for three days and then characterized in more detail.

### 2.7 Immunohistochemistry of skin substitutes

For the immunohistochemistry (IHC) analysis the samples were fixed in 4% (w/v) paraformaldehyde for 60 min and then washed with DPBS. The samples were either directly stained for analyzing the topography from top view using the described protocol or first cut into 150 µm layers using the VT1200 S vibratome (Leica) for the histological characterization (side view of topography). The samples were treated with a permeabilization buffer including 0.1% Triton X-100 for 30 min, then washed with DPBS plus 0.05% Tween20. The samples were then blocked with a 0.2% I-BLOCK^TM^ blocking buffer (Invitrogen^TM^). Overnight the respective primary antibody was added (fibronectin, Invitrogen, Krt5, BioLegend, collagen IV, Invitrogen, laminin-332, Thermo Fisher Scientific). On the next day, the samples were washed again with DPBS plus 0.05% Tween20 and the secondary antibody was added (Alexa Fluor 647, Dianova). After an incubation period of 60 min the samples were washed again. For visualization of cell nuclei a Hoechst-dye (ThermoFisher) was added for 15 min and washed subsequently three times with DPBS plus 0.05% Tween20 for 5 min.

### 2.8 Microscopy

All microscopic images were obtained with the fluorescence microscope BZ-X800 (Keyence) or the cLSM (Zeiss) and analyzed and processed with Zeiss Zen 3.10 (Zeiss) and Fiji (ImageJ).

## 3 Results

### 3.1 GFP-HDFn cells proliferate in GelMA and produce ECM

**Fig. 2:**
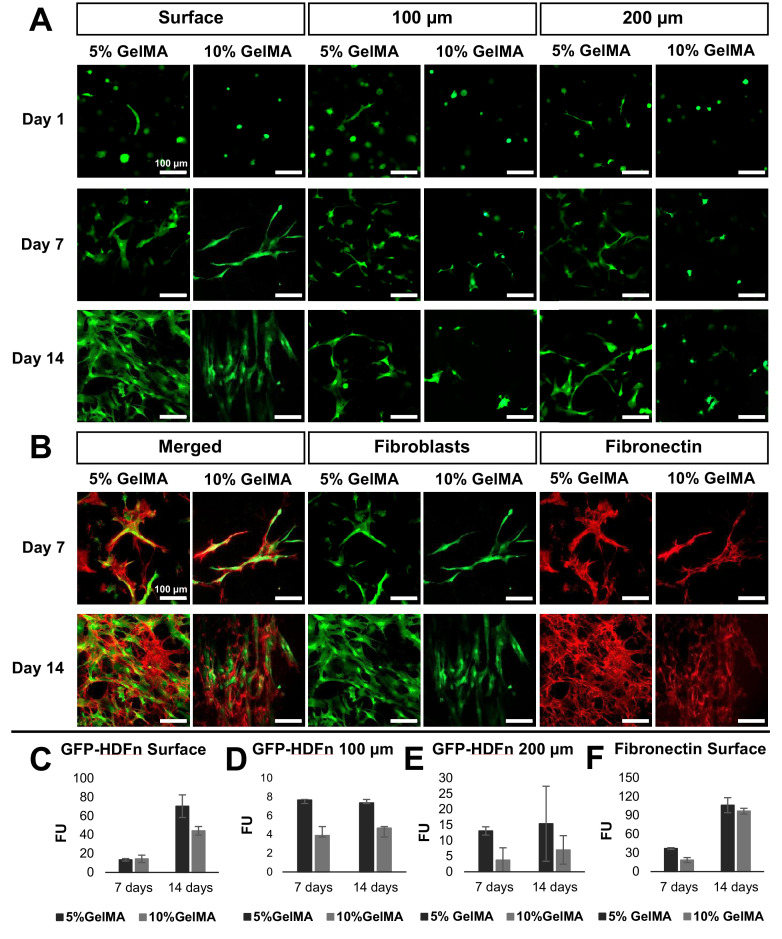
A GFP-HDFn growth in GelMA. The shape of GFP-HDFn were determined on three time points and three locations (surface, 100 µm and 200 µm deep in GelMA. At day 1 first GFP-HDFn cells start stretching inside and on top of the 5% GelMA but no cells in 10% GelMA. At day 7 stretched GFP-HDFn could be detected in 10% and 5% GelMA. GFP-HDFn reach confluency after 14 days on 5% GelMA but still not on 10% GelMA. In 10% GelMA most GFP-HDFn are still spherical after 14 days. (n= 3, for each time point) B Fibronectin production of GFP-HDFn on GelMA after 7 and 14 days (n=3). C-F Fluorescence intensity in fluorescence units (FU) of GFP-HDFn on the surface, in 100 µm and 200 µm depth and fibronectin on the surface after 7 and 14 days. On the surface the FU of GFP-HDFn and fibronectin increase in both concentrations over time, but inside the GelMA there are nearly no changes between 7 and 14 days (n=3).

One of the major challenges in tissue engineering is diffusion of nutrients into deeper tissue layers and removal of metabolic by-products [40]. Cells must be supplied with sufficient nutrients to fulfill their function and to survive. Therefore, two gelatin methacrylate (GelMA) concentrations (5% and 10%) were examined to determine which GelMA concentration provides pore sizes that allow human neonatal dermal fibroblasts expressing green fluorescent protein (GFP-HDFn) to spread, proliferate, and survive within the artificial tissue. GelMA is photo cross-linkable in combination with a photo initiator like lithium phenyl (2,4,6-trimethylbenzoyl) phosphinate (LAP). LAP is not only activated by ultraviolet (UV) light but also at a wavelength of 405 nm [41]. GFP-HDFn were analyzed concerning their proliferation, spreading and fibronectin production in the different GelMA matrices (Fig. 2 A, B). For quantification, the fluorescence units (FU) of the images in Fig. 2 are analyzed (Fig. 2 C–F). A 5% GelMA gel loaded with GFP-HDFn is compared to a 10% GelMA gel both on the surface and in depths of 100 μm and 200 μm within the gels. On 5% GelMA-gels, the GFP-HDFn proliferate and reach confluency at day 14 and produce a high amount of fibronectin. The FU values of GFP-HDFn are 13 at day 7 and 70 at day 14 (Fig. 2 C). The FU values for fibronectin production are 37 at day 7 and 106 at day 14 (Fig. 2 F). In the 10% GelMA-gels GFP-HDFn also spread, proliferate, and produce fibronectin, but do not reach confluency at 14 days (Fig. 2 B). The FU value for 10% GelMA are 14 at day 7 and 44 at day 14 (Fig. 2 C). The FU value for fibronectin production on 10% GelMA are 18 at day 7 and 97 at day 14 (Fig. 2 F). As shown in Fig. 2 B, confluency is not achieved, resulting in a lower FU than with 5% GelMA. However, in depths of 100 µm and 200 µm GFP-HDFn proliferate and spread only in 5% GelMA, not in 10% GelMA. On the first day, all cells are spherical, and only single cells begin to spread in 5% GelMA. In 10% GelMA, no GFP-HDFn were widely spread during the 14-day cultivation period. Furthermore, the FU value of 10% GelMA remained unchanged after day 7 until day 14 as well as at the depths of 100 µm and 200 µm (Fig. 2 D, E). In summary, GFP-HDFn proliferate, spread, and produce more fibronectin in 5% GelMA, which indicates a more physiologically suitable hydrogel composition. Therefore, the next experiments in this study were conducted using 5% GelMA.

### 3.2 Precise 3D printing of a template to mimic the DEJ topography

**Fig. 3:**
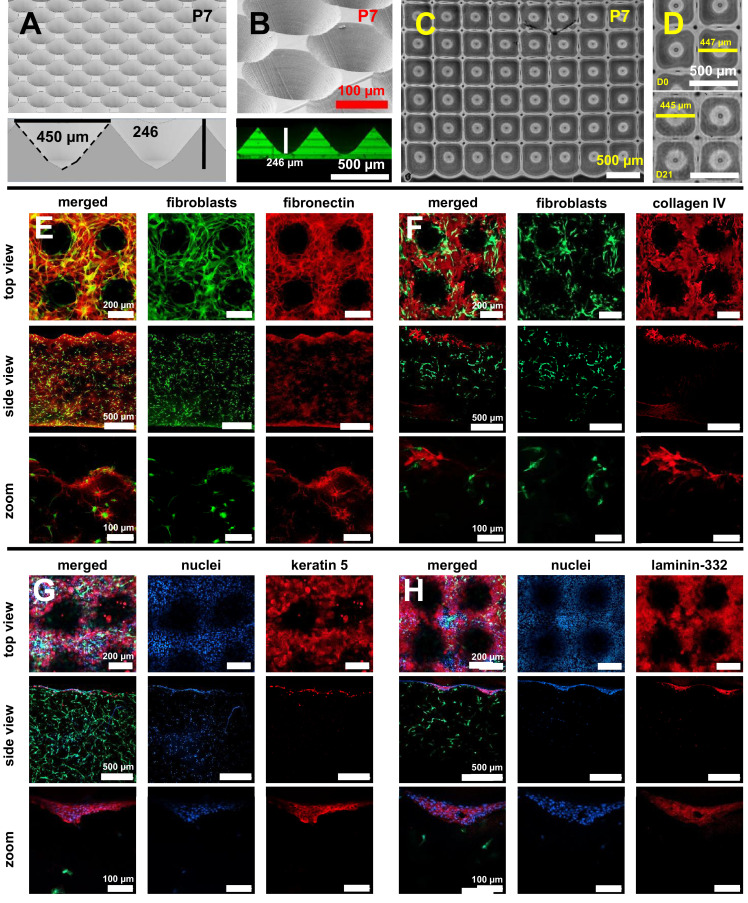
A-C Pattern fabrication from CAD to 2PP print to GelMA. A CAD-Design of P7. B SEM of 2PP-printed P7 and LSM cross-section of P7 including dimensions, (material: UpPhoto, n=3) C Microscopy image after 2. mold of P7 (translation to 5% GelMA). D Microscopy images of 5% GelMA from P7 after production (D0) and after incubation under cell growth conditions for 21 days (D21). The width does not change over the period under cell culture conditions (n=2). E-H 14 days incubation with GFP-HDFn (green) mixed with 5% GelMA. In the first row is the top view, in the second the histological cut and in the third row the 20x magnification of the previous image (cross-section = 150 µm; each immune staining n=2; total DEs n=16 from these DEs eight were used for N/TERT-1 keratinocyte co-culture). E Fibronectin detection on GelMA DE and in histological cuts. A high amount of fibronectin is detectable (n=4). F Immunohistochemistry (IHC) of ColIV production on the surface of the DE (n=4). G-H N/TERT-1 keratinocytes are seeded on the DE for 3 days (n=8). G IHC of Krt5 production in N/TERT-1 keratinocytes (n=4). H IHC of laminin-332 production of N/TERT-1 keratinocytes after 3 days on DE (n=4).

To develop a 2PP-printed template that replicates the architecture of the skin’s dermal epidermal junction (DEJ), eight different topographies were designed using computer-aided design (CAD) and combined into a single layout (see supplement Fig. S1). The screening plate comprising eight different geometries is essential for further studies to reach physiological rete ridges on the DE after the molding process and tissue remodeling. The screening plate, for example of pattern 7 (P7) includes two conical structures. To prevent the cone from tapering too sharply at a diameter of 450 μm and an angle of 60°, it was truncated at approximately 200 μm, and a new cone with a significantly flatter angle (40°) was placed on top of the previous one. Thus, P7 is 246 μm deep at its lowest point (Fig. 3 A). SEM analysis and LSM images provide insights into the printed topography and the side view of the 2PP-printed samples, allowing for an analysis of their actual dimensions (Fig. 3 B). Characterization of the 2PP-printed patterns shows no significant differences of the obtained 2PP structures compared to the CAD design. The standard deviation of the P7 dimensions for example are SD_depth_= 0.232 µm and SD_width_ = 0.013 (n=3). In summary, all patterns show minimal standard deviation, and the deviation of the individual structures of each individual pattern is ≤0.44%, indicating excellent reproducibility of the individual prints using 2PP (see supplement Fig. S2 and Table S1).

### 3.3 Translation of the 2PP pattern topographies on a GelMA based hydrogel

To generate a DE with the designed topography, at first a protocol for the molding had to be developed. Molding surfaces with GelMA or gelatin is challenging, as these materials adhere to other materials like silicones. So, the use of an agarose hydrogel simplifies the molding process, as gelatin and GelMA cannot adhere to agarose. In the first step, the printed screening plate was molded with 3% agarose (Fig. 1 C, 1. Molding). The resulting negative agarose pattern bed was used to transfer the pattern structure onto the GelMA hydrogel as described in 2.6 (Fig. 1 C, 2. Molding). By simply detaching the GelMA from the agarose, surfaces are molded efficiently. Even the “stitching scars” from the 2PP printing process are still clearly visible in the macroscopic and microscopic analyses even after two molding steps (Fig. 3 B and see supplement Fig. S3). The macroscopic analysis clearly shows that no cracks occurred during molding, and all patterns were completely transferred (Fig. 3 C and see supplement Fig. S3). To verify the molding quality, the widths of all patterns are measured (see supplement Table S1). Additionally, the GelMA pattern stability was measured after a 21 day cultivation period under cell culture conditions (Fig. 3 D). The width of P7 directly after fabrication (day 0) was 440 - 450 µm. In comparison, after 21 days the width was still 440 – 450 µm, resulting in no significant dimension changes. The depths of the patterns were not further analyzed.

### 3.4 GFP-HDFn proliferate in the GelMA screening plates and start remodeling the pattern structure

In the next step, a DE containing all patterns (P1 – P9) on its surface was produced as described above, but with the addition of GFP-HDFn, and cultivated for 14 days. GFP-HDFn generated a high amount of fibronectin and started to remodel the surface of the DE (see supplement Fig. S4). P1 to P6 and P8 were strongly modified by the GFP-HDFn, so that only a few invaginations (P1, P2, P3, P8) or no invaginations (P4, P5, P6) were visible (see supplement Fig. S4). Characterization of P7 shows that the cone structure is preserved but the invaginations are massively reduced in size and lost almost 50% of their previous size (Fig. 3 E). Nevertheless, P7 reaches physiological parameters after remodeling. Based on these results, P7 was used for further studies. The GFP-HDFn are widely spread in the DE and after the cultivation period fibronectin is detectable in all layers and ColIV as a layer on the patterned surface (Fig. 3 E, F). ColIV could not be detected inside the DE. This indicates that the GFP-HDFn were supplied with sufficient nutrients up to a layer depth of 1500 µm and the viability of the cells indicate that the metabolic by-products which would influence the viability were also removed. The result of the screening plate indicates that the topographic structure of P7 is in the physiological range even after 14 days in culture with GFP-HDFn and still possesses cavity widths of 200 – 300 µm and depths of 80 – 110 µm. The decisive factor here is that different invaginations show different depths, just as they occur in nature. In summary, a DE with ECM-producing fibroblasts localized in a structured GelMA pattern that mimics the topography of physiological human DEJ (n=8) has been developed and was used in a proof-of-concept to form an epidermis like structure including N/TERT-1 keratinocytes on top.

### 3.5 N/TERT-1 keratinocytes adhere on DE with DEJ pattern and form a monolayer

In a final validation step, N/TERT-1 keratinocytes were applied to the previously developed dermis model to confirm its functionality. For this approach, N/TERT-1 keratinocytes were seeded onto the DE derived from P7 after 14 days and co-cultured for three days (n=8). Two important factors were considered in more detail. Firstly, it was investigated whether the N/TERT-1 keratinocytes adhere to the DE and form a monolayer, and secondly, whether the N/TERT-1 keratinocytes express the basal keratinocyte markers Krt5 and laminin-332. N/TERT-1 keratinocytes adhered to the DE and formed monolayers (Fig. 3 G-H). The patterns were confluently covered with Krt5-positive N/TERT-1 keratinocytes (Fig. 3 G). Inside the dermal part of the skin model the GFP-HDFn are Krt5 negative. Antibody staining reveals a monolayer of laminin-332 positive N/TERT-1 keratinocytes (Fig. 3 H). The cells are located both on the plateau and within the invaginations. The cross-section shows a distinct red line, indicating the expression of laminin-332. This is further clarified in the magnified view. In summary, we successfully developed a DE with physiological topography and properties. In this proof-of-concept, N/TERT-1 keratinocytes adhere to the patterned DE and do not differentiate within three days, since they form a Krt5-positive monolayer.

## 4 Discussion

Mimicking the complex architecture of the DEJ is a major challenge in skin tissue engineering. In this study, a novel approach to mimic the 3D-topography of the dermis is presented. In this process, 2PP enables the fabrication of microstructures in the form of a 3D topography with nanometer-scale precision [[16], [32], [33], [34]], thereby generating physiologically relevant DEJ properties that accurately replicate the *in vivo* morphology. Compared to models like Phenion® [19], Episkin® [18], or EpiDerm™ [17], the introduced DE enable DEJ-specific customization by modifying the rete ridge geometries in CAD prior to 3D-printing. This recently developed method is important for modeling age-related flattening of the rete ridges and for novel disease models such as psoriasis and epidermolysis bullosa, that affect the morphology of the DEJ [7,10,11]. The accuracy of the printed topography was confirmed by comparing the CAD designs with the 2PP printed structures using confocal microscopy. The deviations remained within a range of ≤0.44%, highlighting the accuracy and reproducibility of the 2PP method and confirming the relevance of this technique for tissue engineering purposes.

Importantly, the DE, established as 3D pattern translated to a GelMA gel using two consecutive molding steps, supported the interaction of GFP-HDFn. Fibroblasts migrated from the GelMA hydrogel into the invaginations and initiated tissue remodeling, resulting in the selection of only P7 as a physiological model which remained stable over the culture period of 14 days despite fibroblast infiltration. Although mechanical testing was not part of this initial study, the observed cellular organization and tissue morphology suggested that the DE exhibits a certain degree of strength under cell culture conditions. Furthermore, the DEs showed optical transparency for imaging and biocompatibility, which is consistent with previous studies on GelMA cell-permeable matrices for 3D tissue culture [42]. GFP-HDFn produced not only a high amount of fibronectin in the DE, additionally, they also formed a monolayer of collagen IV (ColIV) on the surface. ColIV is one of the main ECM components that interact in the DEJ with basal keratinocytes in the basement membrane and forms hemidesmosomes in interaction with laminin-332 and collagen XVII [8,9].

Since the DE derived from P7 maintained its structure for 14 days of fibroblast cultivation, N/TERT-1 keratinocytes were seeded on the DE and cultured for 3 days. N/TERT-1 keratinocytes formed a basal monolayer three days after seeding, which was detected by Krt5 expression and exhibited a topography with physiological dimensions. Furthermore, laminin-332 positive N/TERT-1 keratinocytes were detected, indicating an active connection between the growing epidermis and the dermis [8,9].

For the last 25 years, skin tissue engineering successfully generated skin substitutes, where only some of these developed rete ridges and/or included clusters of β1-integrin positive keratinocytes [[43], [44], [45]]. To our knowledge, other mimicked physiological topographies by 3D-printing methods, for example electrospinning, and transfer to PDMS patterns, feeder layer systems or 3D-bioprinted scaffolds [[28], [29], [30],^46^]. In comparison to these studies our recently developed method allows for higher resolution, for higher reproducibility and for usage of our GelMA bioinks, enabling improved interaction between an artificial model of dermis and epidermis and minimizing the need for a feeder layer system [[28], [29], [30],^46^].

## 5 Conclusion

This work introduces a novel micropatterned GelMA-based dermis equivalent (DE) that integrates a topography with physiological dimensions using a high-resolution two-photon polymerization printing technique. Overall, the development of a patterned template using 2PP for a novel DE represents an innovative step forward in the field of skin tissue engineering and provides an advanced model for studying skin physiology and pathology. Special care should be taken to select larger invagination dimensions. The intended diameter should not be less than ∼200 µm and the 3D feature should be increased to ∼200% of the intended size to counteract fibroblast remodeling.

The model not only simulates important topographical features of the natural skin but also assists in the formation of N/TERT-1 keratinocyte monolayers and supports the co-culture of dermal fibroblasts and N/TERT-1 keratinocytes. Nevertheless, an immortalized cell line such as N/TERT-1 keratinocytes cannot reflect the differentiation properties of primary basal keratinocytes and are not suited for generating a full-thickness skin model but give a first impression how primary human epidermal keratinocytes could adhere and proliferate on the DE. However, despite these promising results, there are limitations to consider. The current model does not include melanocytes, immune cells or vascularization [1,24]. In addition, while N/TERT-1 keratinocytes provide consistency and reproducibility, they cannot fully reproduce the behavior of primary keratinocytes and/or β1-integrin-positive epidermal stem cell clusters [38,47,48], especially in response to inflammatory or wound healing stimuli [25,37]. To identify potential epidermal stem cell niches, in future studies β1-integrin-, LGR6-, and LRIG1-positive primary human epidermal keratinocytes (HEKs) need to be identified [[36], [37], [38],^47^]. Such clusters are essential for epidermal renewal and long-term functionality and are rarely observed in conventional flat skin models[16-18,43,44]. Follow-up studies under air-liquid interface conditions with primary HEKs will be crucial to evaluate complete epidermal layering and barrier formation. Nevertheless, N/TERT-1 keratinocytes are suitable for establishing the methodology to produce a functional DE. Future studies will aim to integrate additional cell types and evaluate long-term behavior.

The ability to customize the geometry of the DEJ makes the system extremely suitable for modeling aging, wound healing and DEJ-related skin diseases. Overall, this platform represents a significant step towards the development of next-generation skin equivalents that are both structurally and functionally bio-mimicking.

## CRediT authorship contribution statement

**Sebastian Schröder:** Writing – original draft, Project administration, Conceptualization. **Holger Rothe:** Methodology, Conceptualization. **Elisabeth Rehbein-Bode:** Methodology, Conceptualization. **Doris Heinrich:** Writing – review & editing. **Nicole Teusch:** Writing – review & editing, Supervision. **Nadine Brandes:** Writing – review & editing, Supervision, Project administration.

## Declaration of competing interest

The authors declare that they have no known competing financial interests or personal relationships that could have appeared to influence the work reported in this paper.

## Data Availability

The authors do not have permission to share data.

## Appendix Supplementary materials

Image, application 1
