# Supplementary material for: Novel dermis niche equivalent based on a 3D-structure fabricated by 2-photon polymerization
Source: Biomater Biosyst. 2026 Jul 23;23:100143. doi: 10.1016/j.bbiosy.2026.100143 (PMC13448460; doi:10.1016/j.bbiosy.2026.100143)
Supplement: Supplementary file 1 [file mmc1.docx]

Supplement

For the development of a 2PP-printed pattern template that represents the architecture of the skin DEJ, a topography was designed in Computer Aided Design (CAD) by designing and combining eight geometric structures plus a structure without a pattern. The structures are described as follows: cylinders (P1) and curved cylinders (P2) with a diameter of 150 µm and a depth of 150 µm, followed by cones with a diameter of 150 µm and a depth of 135 µm (P3) and curved cones (P4). Moreover, the dimensions of the cones were increased to obtain the patterns P5 and P6. Enlarging the cone dimensions and using different methods in CAD to place additional curves across the cone structure generated P5 and P6 with a depth of 165 µm and a diameter of 280 µm. For P8, additional curved features were introduced onto the P7 geometry, resulting in the maximum depth of 246 µm is not changed, but the width is reduced to 300 µm (see supp. Figure S1 A). Comparison of the fused CAD pattern with the corresponding 2PP-printed pattern revealed no detectable differences. The stitching “scars” from the 2PP-printing process are clearly visible. These outlined boxes defined by stitching scars are a single field of view in the printing process (Figure S1 B and C). The SEM analysis gives an insight into the printed topography, as well as the side view of the 3D-printed patterns to analyze the actual dimensions (Figure S2). In direct comparison with the calculated dimensions from the CAD file (Figure S1 A) to the printed patterns (Figure 2 A), a minimal standard deviation (SD) of 0.079 µm in P4 has been observed. The SDs of the other printed patterns are as follows: P1_SD_ = 0.625 µm, P2_SD_ = 0.505 µm, P3_SD_ = 0.600 µm, P5_SD_= 0.118 µm, P6_SD_= 0.571 µm, P7_SD_= 0.247 µm and P8_SD_= 0.232 µm. All CAD and 2PP-print dimensions are listed in Table S1. In summary, all patterns show minimal standard deviation, and the deviation from the individual structures on each individual pattern is ≤0.44% (P3), indicating excellent
reproducibility of the individual prints using 2PP. The deviation of each single pattern is calculated as follows: P1= 0.42%, P2= 0.37%, P4= 0.06%, P5= 0.07%, P6= 0.35%, P7= 0.1% and P8= 0.09%.

Macroscopic analysis showd that no cracks occurred during molding and the patterns are intact. (Figure S3 A, B). The microscopic images of the patterns are displayed in Figure 3 D. In comparison to 2PP printed studies, the individual patterns are difficult to distinguish macroscopically, but microscopically the cylinders (P1) are well visible as well as the larger cone structures (P7 and P8). The arrow in P9 supports the identification of the single structures. The different widths of the patterns are also detectable when directly compared to each other, for example P7 and P3. All widths are included in Table S1. A stability assay to determine no structural change over time during an incubation period at 37 °C is displayed in Figure S3 C. The width of P7 was analyzed directly after molding and after 21 days incubation. No significant dimension changes could be detected.

Representative fluorescence microscopy images of all patterned GelMA surfaces after 14 days of cultivation are shown in Figure S4. GFP-HDFn (green) and fibronectin (red) were detected on all investigated patterns. While patterns P1–P6 and P8 showed largely continuous cell layers with only minor visible topographical features, the invaginations of P7 remained clearly distinguishable. On the planar control (P9), GFP-HDFn formed a continuous monolayer with uniform fibronectin expression.

**Figure S1 A** CAD construction of single patterns on the screening pattern template (P1 - P9) including their dimensions. **B** Pattern fusion in CAD top view with included arrow on P9 directing to P1. **C** Fluorescence microscopy image of the 2PP-printed screening plate (material: UpPhoto) scale bar: 3000 µm.

**280 µm**

**150 µm**

**150 µm**

**135 µm**

**260 µm**

**150 µm**

**135 µm**

**150 µm**

**125 µm**

**280 µm**

**165 µm**

**165 µm**

**246 µm**

**450 µm**

**246 µm**

**300 µm**

**P1**

**P2**

**P3**

**P4**

**P5**

**P6**

**P7**

**P8**

**P9**

**300 µm**

**B**

**C**

**A**

**Figure S2** **Microscopy and statistical analysis of each single pattern.** **A** SEM images with a (material: UpPhoto) 45° tilt (scale bars: 100 µm) and horizontal LSM analysis of all patterns (P1 – P9) scale bars: 500 µm. **B** Statistical analysis of each single pattern depth (n=3) and single measurements (n=3) compared to the calculated CAD dimensions. Standard deviation is as follows P1_SD_= 0.625 µm P2_SD_= 0.505 µm, P3_SD_= 0.600 µm, P4_SD_= 0.079 µm, P5_SD_= 0.118 µm, P6_SD_= 0.571 µm, P7_SD_= 0.247 µm and P8_SD_= 0.232 µm. No significance could be detected.

**P1**

**P2**

**P8**

**P9**

**P3**

**P7**

**P5**

**P4**

**P6**

**100 µm**

**A**

**B**

**500 µm**

**45° tilt**

**horizontal**

**45° tilt**

**horizontal**

**45° tilt**

**horizontal**

Depth max [µm]

|  | CAD calculation | Average of 2PP-prints |
| --- | --- | --- |
| P1 | 150 | 149.694 |
| P2 | 135 | 135.177 |
| P3 | 135 | 134.771 |
| P4 | 125 | 125.344 |
| P5 | 165 | 165.049 |
| P6 | 165 | 165.178 |
| P7 | 246 | 245.816 |
| P8 | 246 | 246.178 |

**Figure S3** **GelMA Screening pattern images. A** Macroscopy image of 5% GelMA pattern after two consecutive molding steps (2PP-printed pattern template → 3% agarose → GelMA) (n= 2). **B** Microscopy image of complete screening pattern. scale bar: 3000 µm. **C** Detailed microscopy images of each single pattern directly after molding. The stitching scars from the printing process are slightly visible in P1 - P8 and can be clearly recognized in P9. All patterns are molded. scale bar: 500 µm.

**500 µm**

**B**

**P2**

**P3**

**P4**

**P1**

**P9**

**P5**

**P8**

**P7**

**P6**

**A**

**C**

**Figure S4 GFP-HDFn remodel pattern surfaces.** GFP-HDFn in green and fibronectin in red (AF647). P1–P6 and P8 show almost no invaginations, and the previous patterns are no longer discernible. Only P7 clearly shows the pre-established pattern and has not been overgrown completely by the GFP-HDFn.
(n = 3) On P9, the planar reference, GFP-HDFn formed a monolayer and expressed fibronectin.

**Fibronectin production on patterns P1 – P9**

**P2**

**P3**

**P4**

**P1**

**P9**

**P5**

**P8**

**P7**

**P6**

**500 µm**

| **Pattern** | **Type** | **CAD_h_** | **CAD_w_** | **2PP-printed_d_** | **SD printed_d_** | **2PP-printed_w_** | **SD printed_w_** | **GelMA_w_  (wo GFP-HDFn)** | **GelMA_w_  (w GFP-HDFn)** |
| --- | --- | --- | --- | --- | --- | --- | --- | --- | --- |
| **P1** | cylindrical | 150 µm | 150 µm | 150 µm | 0.625 µm | 150 µm | 0.337 µm | 145 - 150 µm | - |
| **P2** | cylindrical + round edges | 135 µm | 260 µm | 260 µm | 0.505 µm | 260 µm | 0.272 µm | 250 - 260 µm | - |
| **P3** | cones | 135 µm | 200 µm | 150 µm | 0.600 µm | 200 µm | 0.159 µm | 140 - 150 µm | - |
| **P4** | cones + round edges | 125 µm | 200 µm | 150 µm | 0.079 µm | 200 µm | 0.244 µm | 141 - 150 µm | - |
| **P5** | cones + round edges | 165 µm | 280 µm | 280 µm | 0.118 µm | 280 µm | 0.123 µm | 265 - 280 µm | - |
| **P6** | cones + round edges | 165 µm | 280 µm | 280 µm | 0.571 µm | 280 µm | 0.083 µm | 270 - 280 µm | - |
| **P7** | cones | 246 µm | 450 µm | 450 µm | 0.247 µm | 450 µm | 0.076 µm | 440 - 450 µm | 200 - 250 µm |
| **P8** | cones + round edges | 246 µm | 300 µm | 300 µm | 0.232 µm | 300 µm | 0.013 µm | 290 - 305 µm | - |
| **P9** | planar | 0 µm | - | - | - | - | - | - | - |

**Table S1** Calculated, printed and GelMA molded Pattern dimensions including standard deviations (SD).
